# Effect of latitudinal gradient and impact of logging on genetic diversity of *Cedrela lilloi* along the Argentine Yungas Rainforest

**DOI:** 10.1002/ece3.336

**Published:** 2012-09-28

**Authors:** Maria V Inza, Noga Zelener, Luis Fornes, Leonardo A Gallo

**Affiliations:** 1 Instituto de Recursos Biológicos, INTA Castelar-CNIA. N Repetto y De los Reseros s.n. HurlinghamCP 1686, Buenos Aires, Argentina; 2 EEA INTA FamailláRuta 301 km 32, CP 4132, Famaillá, Tucumán, Argentina; 3 EEA INTA BarilocheParaje Villa Verde s/n Ruta 237, CP 8400, S. C. de Bariloche, Río Negro, Argentina

**Keywords:** AFLP, *Cedrela lilloi*, conservation genetics, genetic variation, latitude, logging

## Abstract

*Cedrela lilloi* C. DC. (cedro coya, Meliaceae), an important south American timber species, has been historically overexploited through selective logging in Argentine Yungas Rainforest. Management and conservation programs of the species require knowledge of its genetic variation patterns; however, no information is available. Molecular genetic variability of the species was characterized to identify high-priority populations for conservation and domestication purposes. Fourteen native populations (160 individuals) along a latitudinal gradient and with different logging's intensities were assessed by 293 polymorphic AFLP (amplified fragment length polymorphism) markers. Genetic diversity was low (Ht = 0.135), according to marginal location of the species in Argentina. Most of the diversity was distributed within populations (87%). Northern populations showed significant higher genetic diversity (R^2^= 0.69) that agreed with latitudinal pattern of distribution of taxonomic diversity in the Yungas. Three clusters were identified by Bayesian analysis in correspondence with northern, central, and southern Yungas. An analysis of molecular variance (AMOVA) revealed significant genetic differences among latitudinal clusters even when logging (Φ_RT_ = 0.07) and unlogging populations (Φ_PT_ = 0.10) were separately analyzed. Loss of genetic diversity with increasing logging intensity was observed between neighboring populations with different disturbance (Φ_PT_ = 0.03–0.10). Bottlenecks in disturbed populations are suggested as the main cause. Our results emphasize both: the necessity of maintaining the genetic diversity in protected areas that appear as possible long-term refuges of the species; and to rescue for the national system of protected areas some high genetic diversity populations that are on private fields.

## Introduction

The Yungas, a subtropical montane rainforest from Northwest of Argentina (NWA), is one of the sites of highest biodiversity in this country, especially on the Upper Bermejo River Basin (UBRB). It is known that Yungas plays an important role in providing many environmental services such as watershed protection. This ecosystem is between 400–3000 meters of elevation, and it is located on the southernmost limit of the Neotropical Cloud Forests, a major system in Latin America (Brown et al. 2001). It occurs along a narrow and discontinuous belt throughout the provinces of Salta, Jujuy, and Tucumán in Argentina from 22º to 28º 15′ S. The Yungas has been ecologically subdivided into northern, central, and southern sectors according to the north–south orographic pattern of mountain ranges of Sierras Subandinas and Sierras Pampeanas that precede, from east to west, the Andean Cordillera in Argentina (Brown et al. 2001). The northern sector covers the Sierras de Santa Victoria and Zenta; the center sector covers the Sierras de Lumbrera, Santa Barbara, Centinela, and Maíz Gordo; and the southern sector covers the Sierras de Metán, Aconquija y Medina (De la Sota 1972; Brown et al. 2001). These latitudinal sectors are separated by Chaco Serrano and show a clear decrease in taxonomic diversity with increasing latitude that has been principally associated with less benign weather conditions toward southern Yungas (De la Sota 1972; Cabrera 1994; Morales et al. 1995; Brown et al. 2001; Juárez et al. 2007). In addition, it has been proposed that the climatic history of the region with glacial refuges in northern Yungas during the Pleistocene (Pennington et al. 2000) would have contributed to configuring the current pattern of biological diversity revealed from the geographic distribution of endemic species (Brown et al. 2001, 2006).

The overexploitation of Yungas along the second half last century has been particularly alarming and has caused the habitat forest disturbance (Grau and Brown 2000; Brown and Pacheco 2006). In NWA, logging began after the arrival of the railway in Tucumán in 1876. This promoted commercial development of activity, which was expanded toward the northern region in later decades, while the railway was being extended. Since 1960, mechanization led to forestry overexploitation of Yungas. One of the most affected species was the most valuable timber species of cedar, *Cedrela lilloi* C. DC. (cedro coya), Meliaceae (Minetti 2006; Rivera 2006), Figure 1. Thus, in a regional context of unsustainable management and lack of control and transparency of local market, continuous selective logging for 50 years led to habitat disturbance of accessible forests (Grau and Brown 2000). Presently, forestry focuses on UBRB, mainly in Salta (SAyDS 2007). *C. lilloi*, recently proposed as *Cedrela angustifolia*, is restricted to South America with distribution through warm and humid environments from southern Ecuador to Bolivia and northern Argentina (Pennington and Muellner 2010). As a result of selective logging, trees of high commercial value are now rarely found suggesting genetic erosion of the species. *C. lilloi* has been categorized as ‘endangered’ by IUCN (2011). However, some undisturbed populations still persist in inaccessible and protected areas in Argentina (Minetti 2006). Therefore, it is urgent to enhance conservation status, as well as develop sustainable forest management in remaining populations of protected areas and private fields.

**Figure 1:**
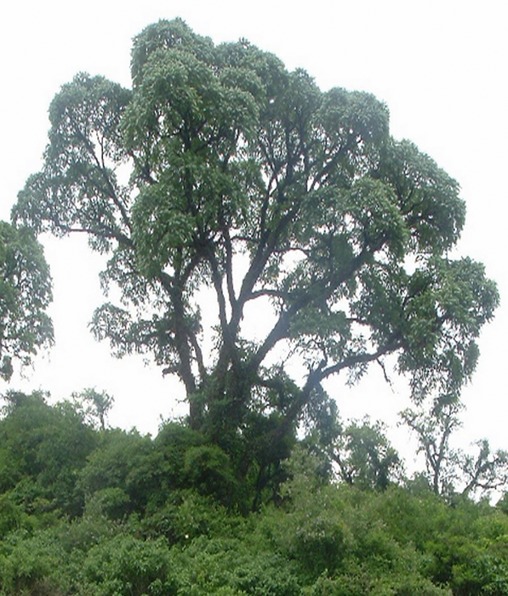
*Cedrela lilloi* in Argentine Yungas Rainforest. Photograph: M.Sc. María Virginia Inza.

Selective logging of timbers trees leads to habitat modification and forest disturbance. Effective size of populations decreases and spatial isolation among remnant trees increases with direct implications to reproductive success of species (Young et al. 1996). Genetic effects are reduction in gene flow, loss of genetic diversity at populations and species, changes in genetic structure, genetic drift, and inbreeding (Young and Boyle 2000). In addition, habitat disturbance affects diversity, abundance, and behavior of pollinators with changes on mating patterns of species (Aizen and Feinsinger 2002; Ward et al. 2005). Several findings have demonstrated that overexploitation of value timbers puts genetic diversity in risk (Hall et al. 1994, 1996; Lee et al. 2002). In *Swietenia* species, a loss of rare alleles (White et al. 1999) and a decrease in heterozygosity (Gillies et al. 1999; André et al. 2008) have been reported. Moreover, in *C. fissilis* populations, an increase in genetic structure (Kageyama et al. 1998) and inbreeding (White et al. 1999; Kageyama et al. 2004) were detected, thus suggesting similar risks in overexploited *C. lilloi* populations.

Genetic diversity is essential to ensure evolutionary processes of adaptation of forest genetic resources to changing environmental conditions and social requirements (Young et al. 1996; Bawa and Dayanandan 1998). Fortunately, intraspecific genetic variation is having a growing acceptance as a valuable tool for designing priority areas for conservation of endangered species (Yanchuk 2001; Gallo et al. 2009). Nevertheless, despite the importance of *C. lilloi* and threat status, no such information is available in Argentina or elsewhere in South America. Because the microsatellites developed successfully in *C. lilloi* are few (Tarnowski 2010) for studies of genetic variation in the species, we performed this study from amplified fragment length polymorphism (AFLP; Vos et al. 1995). This technique requires no previous sequence knowledge, can detect numerous loci per assay, and has been used widely to estimate genetic variation in forest species (Cavers et al. 2003; Lowe et al. 2003; Zelener et al. 2005; De la Torre et al. 2008). In order to assess the pattern of genetic variation in this species according to latitude and logging intensity, we characterized levels and distribution of genetic diversity in *C. lilloi* populations with different histories of use throughout the natural range of this species in Argentina. Our main hypotheses are that: (1) according to warmth-loving habit to *C. lilloi*, the genetic variation in the species in Argentina should follow a latitudinal pattern with a decrease in genetic diversity with increasing latitude toward less benign weather conditions and (2) severely disturbed populations through logging should have decreased the genetic diversity. The central aim of this work was to identify priority areas for conservation and use of the species in Argentine Yungas Rainforest as potential variability sources for domestication, breeding, and conservation programs.

## Materials and methods

### Study species

*C. lilloi* is a canopy, long-lived and deciduous tree species (30–40 m) that occurs along Argentine Yungas between 900–2500 meters of elevation in the upper level of montane rainforest and the temperate montane forest. The populations of this species show trees at low density and aggregated distribution with a gap-dependent recruitment (Grau 2000; Grau et al. 2003; Zamora Petri 2006), which is consistent with the spatial distribution and regeneration patterns described for the genus and family in the Neotropics (Patiño Varela 1997; Mostacedo and Fredericksen 1999; Van Rheenen et al. 2004). Little is known about mating system of *C. lilloi*; however, it is suggested that species of *Cedrela* genus behaves as monoecious and predominantly outcrossing, with high self-incompatibility and small unisexual flowers pollinated by small insects (Ward et al. 2005; Aschero 2006). The fruit is a dehiscent capsule and the seeds are wind dispersed during dry season (Grau et al. 2006).

### Population sites and sample collection

**Figure 2:**
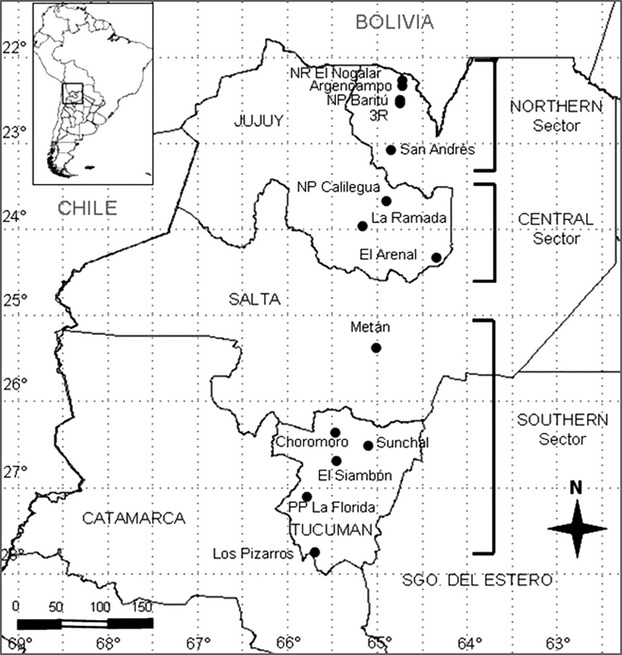
Location of 14 sampled *C. lilloi* populations in northwestern Argentina. This map shows location of populations within three latitudinal sectors of the Yungas. Units are indicated in kilometers.

Young leaves from 160 trees randomly selected were collected from 14 native populations with different histories of use throughout natural range of *C. lilloi* in NWA (Table 1, Fig. 2). For each population, 9–15 trees were sampled according to population size and a minimum distance between trees that was set as 100 meters to avoid collection from family clusters (Gillies et al. 1999). Previous studies in tropical species, such as *Swietenia macrophylla*, *S. humilis,* and *C. odorata,* that also occur at low densities and are at risk have used a similar sampling strategy (Gillies et al. 1999; White et al. 1999; Navarro et al. 2005; De la Torre et al. 2008). To assess the latitudinal pattern of genetic variation in the species populations were grouped in northern, central, and southern sectors, in agreement with orographic and ecological subregions described for Yungas (Brown et al. 2001, 2006). In addition, the effect of logging on genetic variation was studied by sorting populations in three groups according to logging history (disturbance). We consider three indicators to categorize populations by disturbance. First, according to the “logging-free period” to the present, groups were sorted in *disturbed populations* (DP), with present logging or until 20 years without logging; *low disturbance populations* (LDP), with 30–50 years without logging; and *undisturbed populations* (UP), with over 50 years without logging (unlogging). Additionally, information from interviews with forest producers and visual assessments during sampling was added to include the “intensity of logging” and the “history of use” (time under logging, management, accessibility of stands) as others factors that allowed us to achieve a better approximation to disturbance level of logging populations. In protected areas, disturbance was estimated by logging history (prior to its creation) in addition to their age. La Florida Provincial Park (PP, 1936) and Baritú National Park (NP, 1974, highly inaccessible) were classified as UP, whereas Calilegua National Park (1979) and El Nogalar Natural Reserve (NR, 2008) were classified as DP according to history of intense cedar logging. Trees were georeferenced by GPS (Geographic Position System) and identified by individual/population code.

**Table 1:** Genetic diversity and location of 14 *C. lilloi* populations sampled in Argentine Yungas Rainforest. Latitudinal sectors (LS) of Yungas according to Brown et al. (2001) and different disturbance levels of each population are indicated

Population	Code	N	Department, province	Lat. (S)	Long. (W)	Alt. (m)	LS	He (SE)	PL_*P*_	PPL_*P*_ (%)	EB	Disturb. level
Los Pizarros	LPi	10	La Cocha, T	27º 45′	65º 42′	1113	SS	0.049 (0.01)	42	14.3	0	LDP
PP La Florida	FL	9	Monteros, T	27º 07′	65º 47′	1328	SS	0.157 (0.01)	121	41.3	16	UP
El Siambón	LS	15	Tafí Viejo, T	26º 42′	65º 27′	1389	SS	0.057 (0.01)	53	18.1	0	LDP
Sunchal	LSU	9	Burruyacu, T	26º 31′	65º 06′	1468	SS	0.063 (0.01)	57	19.5	1	LDP
Choromoro	LCH	12	Trancas, T	26º 22′	65º 28′	1457	SS	0.056 (0.01)	50	17.1	2	LDP
Metán	ML-MT	10	Metán, S	25º 23′	65º 01′	1102	SS	0.043 (0.01)	36	12.3	0	DP
El Arenal	AL	9	Sta. Bárbara, J	24º 20′	64º 21′	1111	CS	0.059 (0.01)	50	17.1	0	LDP
La Ramada	LLR	12	Ledesma, J	23º 58′	65º 10′	1656	CS	0.071 (0.01)	62	21.2	2	LDP
NP Calilegua	CL	9	Ledesma, J	23º 41′	64º 54′	1642	CS	0.062 (0.01)	52	17.8	1	DP
San Andrés	LSA	15	Orán, S	23º 05′	64º 51′	1744	NS	0.088 (0.01)	90	30.7	5	LDP
Empresa 3R	3R	11	Sta. Victoria, S	22º 32′	64º 45′	1713	NS	0.107 (0.01)	98	33.5	5	DP
NP Baritú	D	15	Sta. Victoria, S	22º 30′	64º 45′	1704	NS	0.168 (0.01)	192	65.5	36	UP
Argencampo	ArD	9	Sta. Victoria, S	22º 20′	64º 43′	1728	NS	0.113 (0.01)	95	32.4	7	DP
NR El Nogalar	ND	15	Sta. Victoria, S	22º 17′	64º 43′	1825	NS	0.119 (0.01)	120	41.0	9	DP

N, sampled size; T, Tucumán; S, Salta; J, Jujuy; SS, southern sector; CS, central sector; NS, northern sector; UP, undisturbed populations; LDP, low disturbance populations; DP, disturbed populations; He, mean expected heterozygosity; SE, standard error; PL_P_, number of polymorphic loci per population; PPL_P_, percentage of polymorphic loci per population; EB, number of exclusive bands.

### DNA isolation and AFLP protocol

Total genomic DNA was extracted from 70–80 mg of dried leaf material following the standard CTAB procedure described by Hoisington et al. (1994) with some minor modifications. DNA quantification was carried out by comparing with known concentration of commercial DNA (Lambda) and integrity was checked by electrophoresis on 0.8% agarose gel containing ethidium bromide (0.002% v/v). The AFLP method followed the procedure described by Khan et al. (2000), and thermal cycling conditions were carried out according to Vos et al. (1995). Briefly, high-quality genomic DNA (250 ng) was digested by two restriction enzymes (*Eco*RI/*Mse*I) and selective amplification was performed using primers having two and four additional nucleotides, respectively (*Eco* + 2/*Mse* + 4), as suggested by Cavers et al. (2003) for *C. odorata*. Twelve AFLP primer combinations from *Eco* + 2 (AA, AC, AG, and AT) and *Mse* + 4 (AACA, AATA, and ACCA) were examined on 16 individuals and those five most clear and polymorphic were selected: *Eco*+AT/*Mse*+ACCA, *Eco*+AC/*Mse*+AATA, *Eco*+AC/*Mse*+AACA, *Eco*+AT/*Mse*+AACA, and *Eco*+AG/*Mse*+ACCA, coded E14M115, E12M107, E12M99, E14M99, and E13M115, respectively. Amplification products were separated by electrophoresis on 6% (w/v) polyacrylamide gels and detected by silver staining (Silver Sequence Promega Biotech, Madison, WI).

### Data analysis

AFLP products were scored as 1 or 0 for presence or absence of bands, respectively, and a binary data matrix was generated. It was assumed that each amplified fragment corresponded to dominant allele (for both heterozygous and homozygous states) and absent fragment corresponded to null homozygote. Only polymorphic bands were included and no homoplasy was assumed. In each gel, samples of known genetic profiles were included as controls. We estimated the total number of polymorphic loci, as well as the number of polymorphic loci (PL_C_) and the percentage of polymorphic loci (PPL_C_) per primer combination.

Genetic diversity (Nei 1973) of populations and total average for the species were estimated as unbiased He from allele frequencies of AFLP data by square root procedure (Bonin et al. 2007) using GenAlEx 6.2 software (Peakall and Smouse 2006). Briefly, frequency of dominant allele was calculated as 1 − the square root of frequency of band absence at each AFLP locus. Other population parameters of genetic diversity were estimated as the number of polymorphic loci (PL_P_), the percentage of polymorphic loci (PPL_P_), and the number of exclusive bands (EB), as well as the total average for the species. In addition, trees that showed exclusive bands at individual level (EBi) were identified, and the number of exclusive bands per individual and the number of trees with EBi per population were recorded.

To examine distribution of genetic variation among and within populations, a hierarchical analysis of molecular variance (AMOVA, Excoffier et al. 1992) was performed using GenAlEx 6.2 software. Genetic differentiation among populations was estimated by Φ_PT_ statistic. Statistical significance of test was assessed from 999 random permutations across full data set. In addition, total genetic diversity (Ht) and its partition within (Hw) and among (Hb) populations, as well as F_ST_, were calculated according to Lynch and Milligan (1994) using AFLP-SURV 1.0 software (Vekemans 2002). Indirect estimates of historical gene flow (N_m_) among populations were obtained from N_m_ = (1−F_ST_)/4 *α* F_ST_, where N_m_ is the number of migrants per generation, and *α* is a correction factor that depends on the number of sampled populations, α = [n/(n−1)]^2^, according to Crow and Aoki (1984). The Bayesian clustering approach was implemented using Structure 2.2.3 software (Pritchard et al. 2000; Falush et al. 2007) and the *K* value, which is the number of genetically homogeneous clusters of populations was estimated. Five replications for each proposed *K* value (from *K* = 2 to *K* = 6) for 500 000 iterations after a 50 000 burn-in period was run. Program setting was performed under no-admixture ancestry model and correlated allele frequencies. We finally selected *K* value that maximized likelihood of data and Δ*K* modal values, and minimized variability of likelihood values, as recommended by Evanno et al. (2005). UPGMA (unweighted pair-group method by arithmetic average) dendrogram showing genetic relationships between populations based on Nei's genetic distance (Nei 1978) was constructed with NTSYS 2.0 software (Rohlf 1998). To examine distortion of dendrogram, a Mantel test between genetic distance matrix from AFLP-SURV 1.0 software and cophenetic matrix was performed and cophenetic correlation coefficient (r) was computed. Genetic distance was tested against geographic distance by Mantel test with 999 random permutations using GenAlEx 6.2 software.

#### Genetic variation and latitude

The effect of latitude on genetic diversity was assessed by He, PPL, and EB for each of three population groups, according to latitudinal sectors of Yungas (Table 1, Fig. 2). This analysis was conducted from three approaches. First, for the total of 14 populations; second, only populations with some disturbance level (LDP and DP) were taken into account; and finally, only undisturbed populations like Baritú (in north) and La Florida (in south) were compared. These last two approaches are to minimize the effect of disturbance. In addition, an AMOVA was conducted to estimate genetic variation among latitudinal sectors. It was performed for each of the three mentioned approaches. Total genetic variation was partitioned among latitudinal sectors (Φ_RT_), among populations within sectors (Φ_PR_), and within populations. Additionally, intrapopulation genetic diversity was tested against latitude with a linear regression analysis performed by InfoStat software (2008 version), using He and PPL as dependent variable. This analysis was carried out for the total of 14 populations, for 12 populations with some disturbance level, and for each disturbance group (excluding undisturbed group because it has only two populations).

#### Genetic variation and disturbance

Logging effects on genetic diversity was assessed by He, PPL, and EB for each disturbance group, after grouping populations according to this factor (Table 1) from different approaches. First, only two categories of disturbance, including unlogged population and logged population groups, were considered. The latter group comprised by LDP and DP populations because they are not latitudinally comparable. Second, the three disturbance groups were compared within each latitudinal sector. Finally, only pairs of neighboring populations with different disturbance levels were compared. These last two approaches are to minimize the effect of latitude. An AMOVA was carried out to detect significant genetic differences among disturbance groups. It was performed for each mentioned approach. Genetic variation among disturbance groups was estimated by Φ_RT_ or Φ_PT_ statistics (for comparing by populations pairs). Particularly, under the second approach, several comparisons were performed: (a) among three disturbance groups, (b) between logged (LDP and DP) and unlogged groups (UP), and (c) between DP and LDP groups.

## Results

A total of 592 AFLP fragments were amplified over 160 individuals of *C. lilloi*, of which 293 (49%) were polymorphic among or within examined populations. Primers combinations differed in their ability to detect polymorphism; thus, E13M115, E14M115, and E12M99 were the most polymorphic, with 70, 70, and 65 PL_C_ and 51%, 52%, and 60% PPL_C_, respectively. In contrast, E12M107 and E14M99 were the least polymorphic combinations, with 51 and 37 PL_C_ and 43% and 41% PPL_C_, respectively.

### Genetic diversity

Genetic diversity of the species over all populations was on average He = 0.087 (Standard Error = 0.003) and PL_P_, PPL_P_, and EB were on average 80 (ranged from 36–192), 27.3% (ranged from 12.3–65.5), and 6 (ranged from 0–36), respectively. As a general pattern, genetic diversity of cedar populations decreased with increasing latitude and disturbance level (Table 1). All genetic diversity indices showed that Baritú (UP) from the northern sector had the highest diversity and Metán (DP) toward the south had the lowest. Genetic diversity of northern populations was double and triple compared with central and southern populations with similar and even lower disturbance, excluding La Florida. This population was the exception in the southern sector, because this was the second most diverse population of the region showing genetic diversity levels comparable with north. The EBi index per population showed a similar trend to that observed for EB. Baritú had the highest number of trees showing EBi (with 26 bands in only 7 from 15 individuals). La Florida and El Nogalar had 9 and 8 EBi, respectively, both populations in 4 individuals. The remaining populations showed EBi values from 5–0.

### Genetic differentiation, structuring, and genetic relationships

**Figure 3:**
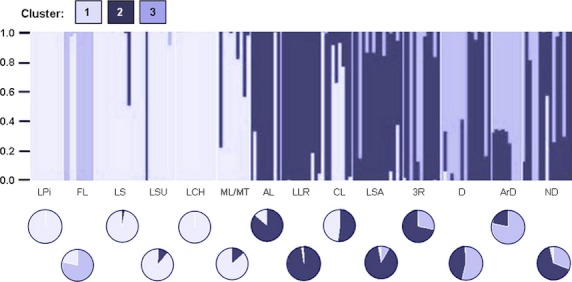
Genetic structure of *C. lilloi* populations inferred from Bayesian clustering method (Pritchard et al. 2000). Populations are ordered from left to right in decreasing order of latitude, and the codes are shown in Table 1. Each vertical bar represents an individual, and the circles represent the percentages of assignment of populations to clusters 1 (light blue), 2 (dark blue), and 3 (blue).

Distribution of total genetic variation by AMOVA revealed that most of total variance is attributable to genetic variation within populations (87%). However, a 13% of the variance was partitioned among populations (Φ_PT_ = 0.130, p ≤ 0.001). When Lynch & Milligan' restriction was applied, total genetic diversity (Ht) was 0.135, most of which was distributed within populations (Hw = 0.119). Genetic diversity among populations was Hb = 0.015 and F_ST_ = 0.115 was similar to Φ_PT_ statistic from AMOVA. Estimated historical gene flow among populations was N_m_ = 1.67. Genetic clusters estimated by Bayesian model was *K* = 3, principally in accordance with the three latitudinal sectors (Fig. 3). All southern populations were assigned to cluster 1 (Los Pizarros, 100%; El Siambón, 97%; Sunchal, 88%; Choromoro, 100%; and Metán, 85%), except for La Florida, with just 22% of individuals assigned to this group. Central populations were assigned mainly to cluster 2 (El Arenal, 85%; La Ramada, 98%; and Calilegua, 52%); and cluster 3 was exclusive of northern Yungas (San Andrés, 9%; 3R, 28%; Baritú, 54%; Argencampo, 78%; and Nogalar, 30%) and La Florida (78%). Moreover, we observed an increase in genetic variability and genetic divergence of populations with decreasing latitude. Thus, populations of southern Salta and Tucumán (except for La Florida) were assigned almost exclusively to one cluster (1), central populations to two clusters (1 and 2), and northern populations to three clusters (1, 2, and 3).

**Figure 4:**
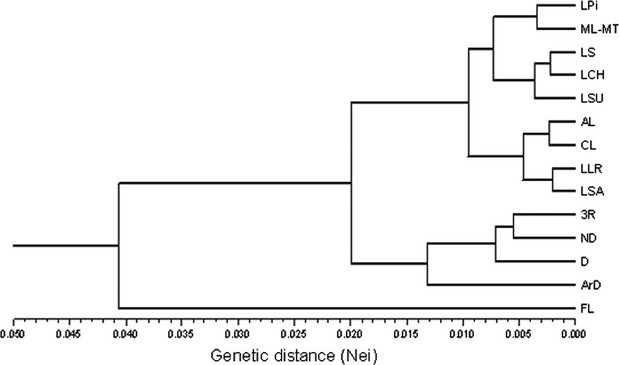
Dendrogram based on Nei's genetic distance showing relationships between *C. lilloi* populations. Population codes are shown in Table 1.

The UPGMA dendrogram resolved them into two groups mainly in correspondence with southern–central and northern edge of Yungas (*r* = 0.90), (Fig. 4). Overall, a decrease in genetic distance among populations with increasing latitude was observed, except for La Florida, which was the most divergent population. In addition, within southern–central group, two subgroups corresponding to populations of Tucumán to southern Salta and populations of Jujuy were identified. In particular, northern population of San Andrés better grouped with central populations. Despite proximity of northern populations, they had greater genetic distances than those observed in south, where more geographic distant populations showed lower genetic distances. These results are very consistent with the clusters identified by Bayesian model.

A positive correlation between geographic and genetic distance was detected (*r* = 0.35, *p* = 0.004), and this increased significantly when undisturbed populations were excluded from analysis (*r* = 0.60, *p* = 0.001). This increase is consistent with the greater genetic distances of La Florida with southern-central populations than with northern populations (separated over 500 km).

### Genetic variation and latitude

**Figure 5:**
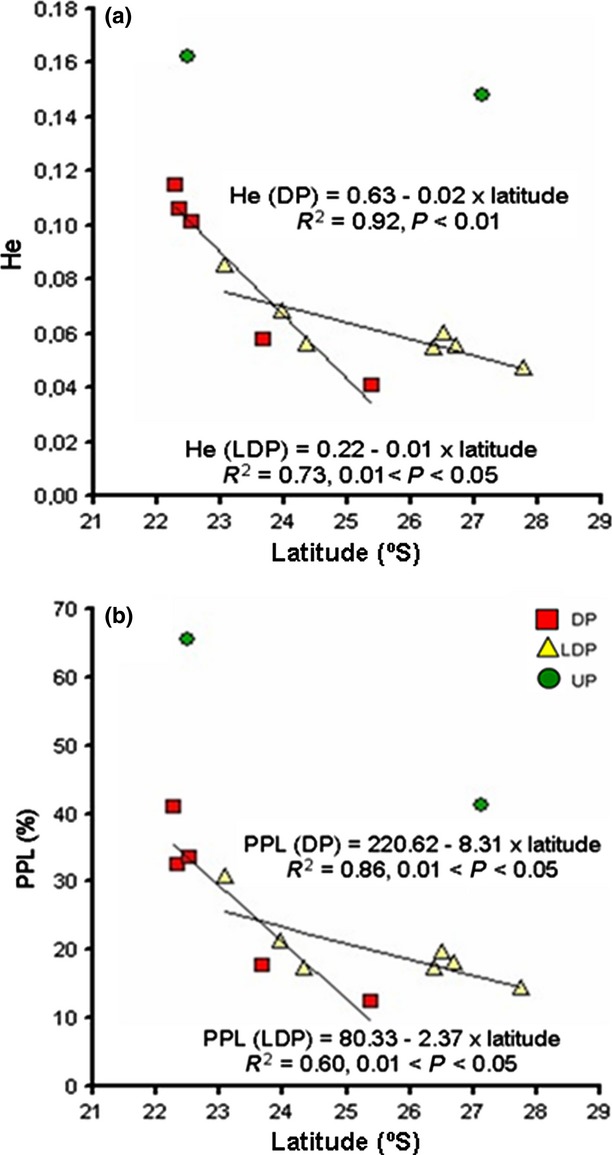
Regression of genetic diversity (A: He and B: PPL) on latitude (ºS) for each different disturbance level (LDP or DP) of *C. lilloi* populations in northwestern Argentina. He, mean expected heterozygosity; PPL, percentage of polymorphic loci per population; LDP, low disturbance populations; DP, disturbed populations.

When genetic diversity was estimated for each latitudinal sector, we clearly observed a higher genetic diversity on the northern sector of Yungas (He = 0.153, PPL = 84.3%, EB = 100). It was followed by southern (He = 0.115, PPL = 50.2%, EB = 26), which is consistent to the high genetic diversity of La Florida. Central Yungas, where all sampled populations are disturbed (LDP and DP), showed the lowest genetic diversity (He = 0.076, PPL = 30.0%, EB = 3). When only populations with some disturbance level were examined, we found a clear decrease in genetic diversity from northern to central Yungas and the same trend although less pronounced from central to southern Yungas. The He was 0.181, 0.104, 0.093 and the PPL was 89.3%, 40.9%, 38.6%, for northern, central, and southern Yungas, respectively. Only for EB, central sector showed a lower value (6) compared with northern (68) and southern (11). For assessment of only undisturbed populations, northern population of Baritú again had higher genetic diversity than southern population of La Florida (Table 1). The AMOVA showed that 5% of the variance is due to the latitudinal factor (Φ_RT_ = 0.05, *p* ≤ 0.001). Genetic differentiation among populations within sectors was Φ_PR_ = 0.09 (*p* ≤ 0.001) and 86% of variance was concentrated within populations. For analysis of only disturbance and low disturbance populations, genetic differentiation among latitudinal sectors increased in 2% (Φ_RT_ = 0.07, *p* ≤ 0.001), which is consistent with the assignment of undisturbed population of Baritú (north) and La Florida (south) to the same group by Structure. Finally, genetic differentiation increased to 10% (Φ_PT_ = 0.10, *p* ≤ 0.001) for only undisturbed populations. Different AMOVA corresponding to three comparative assessments according to latitude are presented in Table 2. Regression of genetic diversity on latitude was no significant when all populations were considered (He, R^2^ = 0.19, p > 0.01; PPL, R^2^ = 0.27, *p* > 0.01). However, it was high, negative, and significant in the analysis of only populations with some disturbance level, with R^2^ = 0.69 (*p* < 0.001) and R^2^ = 0.63 (*p* < 0.01) for He and PPL, respectively. For each disturbance group, relationship between genetic diversity and latitude increased in all cases, except in LDP for PPL (Fig. 5). These results were strongly supported by decreasing of genetic variability observed in north–south direction from both Bayesian cluster analysis and estimation of genetic diversity for latitudinal sectors. Different slopes for DP and LDP could be because these groups are no fully comparable in its latitudinal position. Finally, decline in genetic diversity per unit change of latitude for the PPL was higher than for the He.

**Table 2:** Analysis of molecular variance (AMOVA) according to latitude

Comparative assessment	Source of variation	df	Variance component	% of total variance	Φstatistics	*p*-value
A: total populations	Between latitudinal sectors	2	0.957	5	Φ_RT_ = 0.05	≤ 0.001
	Between populations within latitudinal sectors	11	1.683	9	Φ_PR_ = 0.09	≤ 0.001
	Within population	146	15.717	86	–	–
B: LDP and DP	Between latitudinal sectors	2	1.068	7	Φ_RT_ = 0.07	≤ 0.001
	Between populations within latitudinal sectors	9	1.428	9	Φ_PR_ = 0.09	≤ 0.001
	Within population	124	13.484	84	–	
C: UP	Between populations	1	3.093	10	Φ_PT_ = 0.10	≤ 0.001
	Within population	22	26.525	90	–	–

df, degrees of freedom.

A: total 14 populations; B: only populations with some disturbance level; C:only undisturbed populations.

### Genetic variation and disturbance

The data revealed that logging decreased population genetic diversity. For the total region, unlogged group showed higher genetic diversity (He = 0.193, PPL = 82.9%, EB = 52) than logged group (He = 0.113, PPL = 73.4%, EB = 46). Within each latitudinal sector, the same trend of greater genetic diversity in undisturbed populations was observed. The clearest pattern of genetic diversity decrease with increasing logging was detected in southern Yungas. In this sector, we observed the highest genetic diversity for UP group (He = 0.314, PPL = 82.3%, EB = 22), which was followed by LDP group (He = 0.138, PPL = 55.1%, EB = 11), and DP group had the lowest values (He = 0.086, PPL = 24.5%, EB = 2), for all estimated parameters. At center, LDP group was more diverse (He = 0.245, PPL = 85.2%, EB = 24) than DP group (He = 0.205, PPL = 59.1%, EB = 9). In northern Yungas, UP was the most diverse group (He = 0.200, PPL = 77.7%, EB = 39), while the unique LDP population (San Andrés, He = 0.105, PPL = 36.4%, EB = 8) was less diverse than DP group (He = 0.159, PPL = 68.4%, EB = 31), which could be due to its most southerly location. Finally, comparative analysis of neighboring populations showed clearly that increased logging leads to an important loss in genetic diversity. Populations with lower disturbance level had higher genetic diversity for all compared pairs in three latitudinal sectors, corresponding to Baritú with 3R, Argencampo, and El Nogalar (north); La Ramada with Calilegua (center); and La Florida with El Siambón (south), (Table 1). Effect of logging on genetic diversity of *C. lilloi* populations was confirmed by obtaining a significant genetic differentiation between disturbance groups. For the total region, AMOVA detected that 2% of total variance corresponded to variation between logged and unlogged populations groups (Φ_RT_ = 0.02, p ≤ 0.001), which was low but highly significant. Within each latitudinal sector, a significant genetic differentiation associated with logging only was observed in south (Table 3). Genetic differentiation among three disturbance groups increased to moderate to high (Φ_RT_ = 0.15, p ≤ 0.001), and it became high (Φ_RT_ = 0.22, p ≤ 0.001) in the evaluation of disturbed and low-disturbed populations together as logged group. Only at south, LDP and DP groups showed significant genetic differentiation (Φ_RT_ = 0.05, p ≤ 0.01). Genetic differentiation between pairs of neighboring populations with different disturbance was high in south and low to moderate in north and center (Table 3). The Baritú-3R pair was the unique that showed no genetic differentiation (Φ_PT_ = 0.01, *p* = 0.15).

**Table 3:** Analysis of molecular variance (AMOVA) according to disturbance. Only hierarchical components corresponding to genetic variation among different disturbance levels (Φ_RT_ or Φ_PT_) are indicated. A: within each latitudinal sector; B: pairs of neighboring populations with different disturbance levels

Comparative assessment	Compared disturbance groups or compared populations	Φstatistics	South	Center	North
A: within each latitudinal sector of Yungas	UP, LDP, DP	Φ_RT_	0.15 **	-	0.00 ns
	DP, LDP		0.05 *	0.00 ns	0.02 ns
	Unlogging (UP), logged (DP + LDP)		0.22 **	–	0.00 ns
B: neighboring populations with different disturbance levels	La Florida, El Siambón	Φ_PT_	0.21 **	–	–
	La Ramada, Calilegua		–	0.08 **	–
	Baritú, 3R		–	–	0.01 ns
	Baritú, Argencampo		–	–	0.10 **
	Baritú, El Nogalar		–	–	0.03 *

* *p* ≤ 0.01;

** *p* ≤ 0.001; ns, nonsignificant.

## Discussion

We used 293 AFLP polymorphic loci to evaluate genetic variation in 14 *Cedrela lilloi* populations in Argentine Yungas. According to previous studies, 200 AFLP is an adequate starting point to explore genetic diversity and differentiation (Mariette et al. 2002). Furthermore, estimates with above 250 loci counteract the bias that may occur in measuring genetic diversity from dominant markers (Kremer et al. 2005). The 49% of amplified polymorphic loci was lower than reported for *C. odorata*, in both Peru (98.8%, De la Torre et al. 2008) and Costa Rica (84.8%, Cavers et al. 2003), and this value is consistent with the low genetic diversity that was detected in *C. lilloi*.

### Genetic diversity

Genetic diversity for *C. lilloi* in Argentina is low (Ht = 0.135; Hw = 0,119; He = 0.087) and less than that reported in other AFLP studies for trees (*Pinus pinaster*, Ht = 0.237, Ribeiro et al. 2002; *C. odorata*, Ht = 0.22, De la Torre et al. 2008). However, low genetic diversity has also been observed in other Meliaceae from AFLP, as for Costa Rican populations of *Swietenia macrophylla* (Ht = 0.15, Hw = 0.11, Lowe et al. 2003) and *C. odorata* (Hw = 0.03 to 0.13, Cavers et al. 2003). Genetic diversity under Lynch & Milligan's restriction (Ht) is greater than that obtained for all loci (He), which is consistent with previous studies that have used both approaches (Mariette et al. 2001). Argentine Yungas is on the southernmost edge of Neotropical Cloud Forests, and therefore, it is expected that this ecosystem does not have the richest tropical biodiversity of this system (De la Sota 1972; Brown et al. 2001; Gentry 2001). Additionally, Argentine populations of *C. lilloi* have a marginal position compared with genus and species range in Latin America. Although we have no information about origin of *C. lilloi*, Mesoamerican location of most species of *Cedrela* supports strongly a putative origin of genus in this region (Styles 1981; Pennington and Muellner 2010). Therefore, if Yungas have been more recently colonized by *C. lilloi*, genetic diversity may be lower due to founder effects or population bottlenecks during migration events. Similarly, Cavers et al. (2003) attributed low genetic diversity of *C. odorata* to northern extreme position in Costa Rica. In addition, some biological traits of *C. lilloi* associated with low genetic diversity could be regional distribution, population isolation, and low density (Hamrick et al. 1992; Hall et al. 1994). Finally, it may also reflect human impact leading to genetic erosion of cedar populations (Young et al. 1996).

### Genetic differentiation and structuring

Our findings are similar to that expected for perennial, woody, and predominantly outcrossing species, which maintain most of their variation within populations (Hamrick et al. 1992; Nybom 2004). Population genetic differentiation was similar under different methodologies (Φ_PT_ = 0.130, F_ST_ = 0.115), as suggested by Bonin et al. (2007), and F_ST_ slightly below Φ_PT_ is consistent with studies that have used both approaches (Mariette et al. 2001). Although historical gene flow (N_m_ = 1.67) is above to minimum proposed (N_m_ = 1) to reduce genetic structuring by drift (Young et al. 1996), moderate genetic differentiation between cedar populations and latitudinal sectors of Yungas indicate some restriction in gene flow. This could be attributed to spatial isolation of the high number of sampled populations separated by up to 600 kilometers, which was confirmed from correlation between genetic and geographic distance. Furthermore, natural discontinuity of Yungas, with different mountain ranges and patches or islets in the central sector, might restrict gene flow among populations. It is known that mountains are important barriers to gene flow, especially in insect-pollinated species (Cavers et al. 2003). In addition, habitat disturbance by logging would imply changes on pollination patterns with expected increases in species genetic structure (Aizen and Feinsinger 2002). Reproductive isolation and reduced effective population sizes could be also responsible for the genetic differentiation.

### Genetic variation and latitude

A clear pattern of decline of genetic variability of *C. lilloi* populations with increasing latitude was observed, despite loss of genetic diversity associated with their logging history. This is consistent with latitudinal decrease in biological diversity that has been reported in Neotropical Cloud Forests (Brown et al. 2001; Gentry 2001; Beck et al. 2008) and Argentine Yungas (De la Sota 1972; Cabrera 1994; Brown et al. 2001; Juárez et al. 2007). A significant regression of genetic diversity on latitude in the analysis of only populations with some disturbance level could be explained mainly due to excluding La Florida, which showed greater genetic diversity compared with southern populations. Changes of species diversity with latitude have been generally related to latitudinal environmental gradients (Fischer 1960; Givnish 1999; Beck et al. 2008). *C. lilloi* is distributed through warm and humid environments (Villalba et al. 1992), and therefore, high genetic diversity of northern populations could be explained by large population sizes leading to greater generation and persistence of genetic variability under more benign weather conditions. In contrast, lower genetic diversity of southern populations may be due both to increased selection pressure associated with less benign weather conditions and to location of these populations at the southernmost edge of *Cedrela*'s range. Our results are in strong agreement with latitudinal sectors proposed by Brown et al. (2001) for distribution of biological diversity in Yungas Rainforest (north, center, and south). This pattern follows orographic belts of Andean system in Argentina separated by Chaco Serrano (Cabrera 1994), thus suggesting that gene flow would be limited. Particularly, the greater similarity of San Andrés with central populations could be explained by the more south location of this population in northern sector. Fewer exclusive bands in central Yungas might be attributed to the lowest number of explored trees in this sector, as explained by White et al. (1999). The same discrepancy between northern and southern Yungas was observed for the number of forest species, genera, and families by Morales et al. (1995) in a study at montane rainforest level. In addition, our findings are consistent with results on latitudinal distribution of richness dicot in Argentina, where an increase in the number of species, genera, and families with decreasing latitude was detected and also a significant correlation among these three taxa was reported (Juárez et al. 2007). Thus, a regional pattern of taxonomic diversity is consolidated, now at intraspecific genetic level. Studies of genetic variation of *C. lilloi* in a species context that includes Bolivian populations have further strengthened this Argentine latitudinal pattern (Zelener unpublished). This is very important to develop future management and conservation plans of forest restoration at ecosystem level.

If we consider climate variability as historical processes, latitudinal shifts may also reflect long-term consequences of geological disturbances and refugial isolation (Pianka 1966; Hewitt 2004; Martin and McKay 2004). Our findings are very consistent with the high levels of biodiversity identified on northern edge of Yungas (UBRB), which includes 3000 vascular plants and over 200 tree species (Grau and Brown 2000; Brown et al. 2006). We proposed that northern Yungas may have functioned as a potential long-term refuge for *C. lilloi* during the glacial times of Pleistocene (25,000–12,000 years ago), which are known to have affected the Neotropics (Pennington et al. 2000). Warmth-loving habit to *C. lilloi* suggests that populations could have persisted at lower latitudes during cooling periods, and they have expanded toward southern latitudes during warmer periods. Then, loss of diversity is predicted as a result of demographic and genetic processes, such as population bottlenecks and genetic drift, occurring throughout range expansion. This may explain lower genetic diversity and divergence toward southern Yungas, which is expected in more recently colonized areas (Martin and McKay 2004). Similarly, Pastorino and Gallo (2002) related higher isozyme diversity of *Austrocedrus chilensis* populations of northern Patagonia in Argentina to possible glacial refuges. Greater changes on polymorphic loci compared with heterozygosity are because the loss of any allele leads to change the number of polymorphic loci, whereas only those with higher frequency lead to modify heterozygosity (Lowe et al. 2005). However, although environmental gradients and historical temperature-refuges support highest diversity of northern Yungas, both approaches do not explain high genetic diversity of La Florida.

During Quaternary, glacial cycles had pronounced periods of environmental aridity (Pennington et al. 2000). Therefore, we propose as additional reason that two areas of the region that have the highest rainfall due to their orographic height (Bianchi et al. 2005) may have functioned as historical refuges for *C. lilloi* in Argentine Yungas during extremely dry periods of Pleistocene. They are the UBRB in northern and the wet slopes of Sierra de Aconquija in Tucumán and may explain the high genetic variability of La Florida and populations of northern edge of Salta that are located precisely on the Aconquija system and the UBRB, respectively. This approximation is strongly in agreement with two areas with high concentration of endemic species proposed by Brown et al. (2006) as potential biodiversity refuges in Yungas because of their ecosystem stability in medium- and long-term periods. Genetic similarity between La Florida and northern populations might reflect that both sectors may have been connected during the humidity times of Pleistocene (Morales et al. 1995). Nevertheless, all predictions on climate history and refugial isolation deserve additional phylogeographical studies.

### Genetic variation and disturbance

In addition to latitudinal pattern, results indicate that logging reduces genetic diversity of *C. lilloi* populations, as has been reported for other forest species (Hall et al. 1994, 1996; Kageyama et al. 1998, 2004; Gillies et al. 1999; White et al. 1999; Lee et al. 2002; André et al. 2008). The clearest pattern of decreasing genetic variation with increasing human impact on southern Yungas could be explained for some sector characteristics. First, according to lower initial genetic diversity at higher latitudes, the loss of few alleles in disturbed populations in south could cause greater impact than if it occurs in north. However, if cedar removal in northern Yungas continues at levels that are recorded at present (SAyDS 2007), this would lead to genetic erosion of remnant populations. Second, we could expect greater intensity of use and degradation in Tucumán forests related to the beginning of history logging in this province, which is aggravated by its smaller forested area (Minetti 2006). Finally, all populations in north are in UBRB refuge, while only La Florida in south is in Sierra de Aconquija refuge (Brown et al. 2001). Therefore, discrepancy between disturbed populations and La Florida in south may reflect diversity loss both by recolonization from refuge and by logging. However, in the comparison of neighboring populations, we assumed constant environment and absent of selection pressure. Hence, genetic differences that were detected could be attributed to severe effective population sizes reduction (probably with initial genetic drift processes) in logged populations. The Baritú-3R pair that showed no genetic differentiation is very close and 3R population had a free-logging period for more than 20 years. Our results suggest that cedar logging change demographic structure of populations (reduce size and increase spatial tree isolation), restrict gene flow, and modify species reproductive success leading to loss of genetic variation, first by extraction of adults and then by drift in their descendant populations (Young and Boyle 2000; Degen et al. 2006). Structural changes as reductions in basal area, changes in the distribution of diametrical classes, and minor regeneration related to selective logging were reported for *C. lilloi* populations in Salta (Pinazo and Gasparri 2003).

Loss of genetic diversity process involves several steps (Young et al. 1996; Lowe et al. 2005). First, we suggest that lower exclusive bands at disturbed populations reflect that, in short term, low-frequency alleles (rare alleles) are lost first by gene pool reduction and increased drift in small remnant populations. White et al. (1999) detected higher loss of rare alleles in *S. humilis* populations related to smaller fragment. Second, although the historical gene flow estimated would not still reflect the human impact on Yungas forest, decrease in heterozygosity may suggest that long-term processes, associated with loss of higher frequency alleles by restricted gene flow and drift, are taking place. This is consistent with heterozygosity reductions by logging detected in *S. macrophylla* populations from Mexico to Panama with random amplification polymorphic DNA (Gillies et al. 1999) and from Brazilian Amazonia with microsatellites (André et al. 2008). Progression from initial loss of alleles to a reduction in heterozygosity is directly linked with remnant population size, which depends not only on the time but also on the intensity of impact (André et al. 2008). Genetic diversity reduction may also involve postdisturbance inbreeding. Although inbreeding cannot be detected from dominant marker, it might occur due to low density and aggregated distribution property of *C. lilloi* (Grau et al. 2003; Zamora Petri 2006). Fornes (2006, pers. com) observed *C. lilloi* populations isolated and in small slope areas, thus suggesting crosses among relationship trees. In *C. fissilis* populations, increases in genetic structure (Kageyama et al. 1998) and inbreeding (Kageyama et al. 2004) associated with human impact have been reported in Brazil.

An adequate level of gene flow among remnant trees is a key factor to counteract probable genetic drift (Lowe et al. 2005). It should be considered species characteristics as well as human impact at ecosystem level, particularly on pollinators system. Minute flowers of *C. lilloi* are pollinated by small generalist insects (Ward et al. 2005; Aschero 2006), with more limited foraging ranges compared with specialized insects, thus restricting opportunities for gene exchange (Aizen and Feinsinger 2002). Additionally, during selective logging, majority of removed trees are large reproductive individuals and this might modify abundance flowers of remnant forest leading to reduction pollinator visits. Finally, if we contrast logging effect with latitude effect, results show that genetic variation due to disturbance impact (3–10%) is, at present, lower or equal to genetic variation due to the latitude gradient (10%). Greatest genetic differentiation between La Florida and El Siambón pair (21%) could be attributed to the location of first in a glacial refuge and then should be not considered.

### Implications for conservation and conclusions

Genetic variation of Argentine populations of *C. lilloi* has important implications for its conservation and use. To assign populations to different priority areas in Yungas Rainforest, we evaluated contribution from each one to the total genetic diversity from genetic diversity and uniqueness, as suggested by Petit et al. (1998). We consider Baritú and La Florida as maximum-priority populations for conservation because they have highest diversity and genetic variation that were no present in the rest of the region. Fortunately, Baritú is a National Park nowadays but La Florida is only a Provincial Reserve. As first conclusion, La Florida should be increasing its preservation status and both protected areas used for research purposes. Second, we assign northern cedar populations of Yungas to high-priority conservation area due to their high levels of genetic diversity and differentiation. Thus, promoting the protection status of NR Nogalar (including educational and tourist purposes) is advisable, and turn to sustainable forest management in those populations located on private fields is urgent, especially for San Andrés. Maximum- and high-priority populations are in agreement with the conservation areas of biodiversity of Yungas proposed by Brown et al. (2006): the UBRB in north and Sierra de Aconquija in south. All efforts of regional conservation and germplasm collection for domestication programs should focus here. Legal protection of UBRB was initiated with creation of Biosphere Reserve of Yungas in 2002. In contrast, there are only five Provincial Reserves and one National Park in Sierra de Aconquija, all small and dispersed (SAyDS 2007); therefore, similar conservation framework should protect this area. Finally, eight remaining populations of central and southern Yungas were considered of medium-priority of conservation because of their moderate to low genetic diversity. However, genetic differentiation between center and south and both sectors compare with north suggest an appropriate management of this gene pool. Lowest diversity populations have critical positions that become more important. Los Pizarros is on southern edge of natural range of *C. lilloi* and Metán can act as stepping-stone for gene flow between central and southern Yungas.

To conclude, conservation and use of endangered species should follow some statements. Assessment of genetic variation in species is essential to know the current gene pool to design appropriates conservation strategies. A maximum of populations prioritizing those more diverse and divergent through different environments should be preserved. Conservation only with protected areas is not enough. It is urgent to apply sustainable forest management that will protect populations on private fields. Finally, we expect that genetic and ecological studies about this valuable timber species begin to guide present practices of management and help to take political measurements at regional level, thus showing how knowledge can be translated into effective conservation plans.
